# Necessity and Problems of Cooperation of Mental Health Professionals and Somatically Oriented Doctors in Gender-affirmative Healthcare

**DOI:** 10.1192/j.eurpsy.2025.139

**Published:** 2025-08-26

**Authors:** M. Van Trotsenburg

**Affiliations:** 1 Consultancy genderPRO; 2 Sigmund Freud Private University Vienna, Vienna, Austria

## Abstract

**Abstract:**

Multidisciplinary cooperation is an imperative prerequisite for good clinical practice in general, and for healthcare of gender-variant people in particular. Although self-determination of one’s gender identity is regarded a human right and self-medication in the trans community widespread, gender-affirmative treatment (GAT) with its far-reaching consequences (including reimbursement by social insurances) still is based upon diagnostics – and more importantly – by indication of mental health professionals (MHP). Empathy and affirmative encounter cannot detract from the fact that medical interventions in general cannot be carried out without a diagnosis. It is the assignment and duty of MHPs to make a diagnosis of gender incongruence or gender dysphoria prior to GAT carried out by somatically oriented doctors. This mutual dependency is a constituent of transgender healthcare, not only at the beginning of a transition trajectory but life spanning. Mental and somatic health care are interdependent. Gender affirmative hormonal treatment and its potential side-effects as well as surgical intervention and its potential complications may have large impact on mental health, and vice versa mental health disorders impact medical conditions. MHPs and somatically oriented doctors are socialized and trained in different ways, sometimes complicating understanding. This presentation deals with the needs, peculiarities and obstacles of joint mentoring gender-variant patients from a medical perspective, not only at the beginning of transition but also in times of crisis or in the course of getting older.

**Disclosure of Interest:**

None Declared

